# Naringenin Decreases Invasiveness and Metastasis by Inhibiting TGF-β-Induced Epithelial to Mesenchymal Transition in Pancreatic Cancer Cells

**DOI:** 10.1371/journal.pone.0050956

**Published:** 2012-12-26

**Authors:** Changjie Lou, Fayun Zhang, Ming Yang, Juan Zhao, Wenfeng Zeng, Xiaocui Fang, Yanqiao Zhang, Chunling Zhang, Wei Liang

**Affiliations:** 1 Department of Gastrointestinal Medical Oncology, The Affiliated Third Hospital of Harbin Medical University, Institute of Prevention and Treatment of Cancer of Heilongjiang Province, Harbin, People's Republic of China; 2 Protein & Peptide Pharmaceutical Laboratory, National Laboratory of Biomacromolecules, Institute of Biophysics, Chinese Academy of Sciences, Beijing, People's Republic of China; The University of Kansas Medical Center, United States of America

## Abstract

Epithelial to mesenchymal transition (EMT) promotes cellular motility, invasiveness and metastasis during embryonic development and tumorigenesis. Transforming growth factor-β (TGF-β) signaling pathway is a key regulator of EMT. A lot of evidences suggest that this process is Smad3-dependent. Herein we showed that exposure of aspc-1 and panc-1 pancreatic cancer cells to TGF-β1 resulted in characteristic morphological alterations of EMT, and enhancement of cell motility and gemcitabine (Gem) resistance along with an up-regulation of EMT markers genes such as vimentin, N-cadherin, MMP2 and MMP9. Naringenin (Nar) down-regulated EMT markers expression in both mRNA and protein levels by inhibiting TGF-β1/Smad3 signal pathway in the pancreatic cancer cells. Consequently, Nar suppressed the cells migration and invasion and reversed their resistance to Gem.

## Introduction

Pancreatic cancer is a highly aggressive tumor characterized by early hematogenic and lymphogenic spread and high rates of local recurrence [1]. The failure of the clinical treatment of cancer patients is often attributed to drug resistance and tumor metastasis [2]. However, there remains no effective therapy available to reverse multi-drug resistance and prevent the aggression and metastasis of pancreatic tumor [3], [4], [5]. Thus, a better understanding of drug resistance and metastasis in pancreatic cancer may lead to the development of more effective therapeutic strategy.

Over the past several years, accumulating evidences suggest that epithelial to mesenchymal transition (EMT) plays an important role in tumor progression, metastasis and drug resistance in diverse solid tumors including pancreatic cancer [6], [7], [8]. This process is characterized by loss of epithelial and acquisition of mesenchymal characteristics [9], [10]. During EMT progression, the expression of mesenchymal markers, such as vimentin, N-cadherin and/or fibronectin, is increased, in contract, that of epithelial adhesion molecules, including E-cadherin or/and cytokeratins, is decreased. In addition, the epithelial cells also gain the increased activity of matrix metalloproteinases (MMPs) including MMP2, MMP3, MMP9, which contribute to an invasive and metastatic phenotype [11], [12]. As it is known that tumor cell metastasis is the leading cause of death for cancer patients, the control of EMT process remains a priority for pancreatic cancer therapy.

Previous studies have revealed that transforming growth factor-β1 (TGF-β1) and other growth factors play pivotal roles in driving EMT in the pathogenesis of pancreatic cancer [13], [14], [15]. TGF-β overexpression promotes tumor metastasis in the late stage of tumor [16]. Developing of TGF-β signaling inhibitors has been considered as an attractive way to prevent tumor metastasis. Currently, a number of injectable protein-based TGF-β inhibitors have been developed, including antibodies that disrupt TGF-β ligand binding to the receptor, and oligonucleotides targeting TGF-β1 mRNA [17], [18], [19]. Small molecule inhibitors with a specific target in this signaling pathway have significant advantages in stability and bioavailability compared to macromolecular candidates. Up to date, several small molecule inhibitors have been shown to possess inhibition effects on TGF-β receptor function [20], [21].

Our recent studies have demonstrated that Naringenin (Nar, 4′, 5, 7-trihydroxy flavanone), a natural predominant flavanone, significantly inhibited the transcription of TGF-β1-induced Smad3, and reduced the binding probability of TGF-β1 to its specific receptor TβRII, thus inhibiting the receptor dimerization and the subsequent downstream signal transduction. Moreover, Nar can enhance the anti-tumor effect of doxorubicin to A549 and MCF-7 caner cells by selectively inhibiting the activity of multidrug resistance-associated protein but not p-glycoprotein [22], [23], [24], [25]. These results also demonstrated its potentiality in the treatment of cancer as a TGF-β signaling antagonist.

Here, we try to address whether Nar exerts its anti-metastatic and anti-resistant effects on pancreatic tumor cells by preventing tumor cells EMT through down regulating TGF-β/Smad3 signaling pathway. This study may provide reasonable explanations for Nar's anti-tumor efficacy, and therapeutic benefits in combination of Nar with other anti-cancer drugs.

## Results

### Nar reverses TGF-β1-induced resistance to gemcitabine in aspc-1 cells

**Figure 1:**
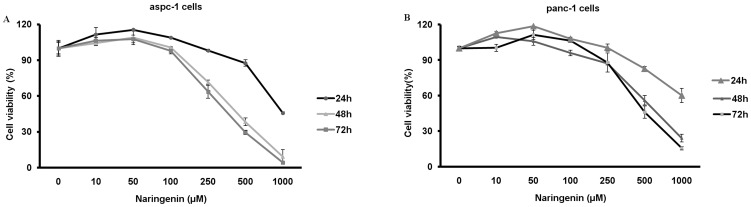
Growth inhibitions of Nar in different pancreatic cancer cell lines. (A) Aspc-1 or (B) panc-1 cells were treated with different concentrations of Nar for 24 h, 48 h and 72 h. Viability of the cells were examined by MTT. Data represent mean ± SD (*n* = 4) from individual experiments points. IC50 were performed using the SPSS software.

**Figure 2:**
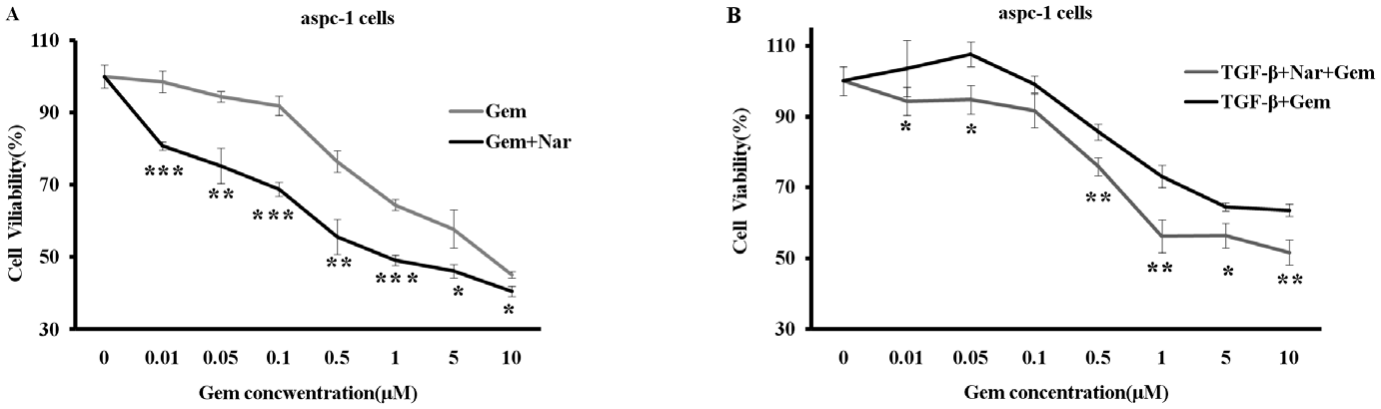
Nar reverses the TGF-β1-induced resistance to gemcitabin in aspc-1 cell line. (A) 50 µM Nar pretreatment for 24 h, followed by combing with different dose Gem for 72 h in aspc-1 cells. (B) Pre-treatment with 5 ng/ml TGF-β1 for 24 h, followed by treatment with various concentrations of Gem in the presence or the absence of 50 µM Nar for next 72 h, the cells viability was evaluated by MTT assay. Each point represents the mean ± SD (*n* = 4) from three independent experiments. *p<0.05;**p<0.01;***p<0.005.

Previous studies showed that aspc-1 and panc-1 pancreatic cancer cells were resistant to gemicitabin (Gem) and had a capability of invasiveness and metastasis [26]. Gem is a common chemotherapeutic drug for patients with pancreatic cancer in clinic. To examine whether Nar enhances sensitivity of these two cell lines to Gem, we determined the potential toxicity of Gem to aspc-1 and panc-1 cells by MTT assay in the presence or absence of Nar. As shown in **Figure 1A and B**, exposure of the aspc-1 and panc-1 cells to Nar for 72 h led to IC_50_ (50% growth inhibitory concentration) values of 347±13.6 µM and 541±11.9 µM, respectively. Nar slightly enhanced cell proliferation up to 100 µM. Furthermore, we measured the cellular viability after 24 h pre-treatment of 50 µM Nar, followed by 72 h treatment with different concentrations of Gem in aspc-1 cells. The IC_50_ values were 5.3±0.4 µM for Gem alone and 2.6±0.4 µM for Gem in the presence of Nar, indicating Nar significantly increases cytotoxicity of Gem to aspc-1 cell (**Figure 2A**). As TGF-β signal pathway plays an important role in promoting resistance of tumor cells to chemotherapy, we examined the effects of Nar on the cytotoxicity of Gem in the cells stimulated with TGF-β1. As expected, TGF-β1 stimulation enhanced aspc-1 cells resistance to Gem compared to the untreated cells (IC_50_>10 µM versus 5.3±0.4 µM **Figure 2B**). Our previous studies have already demonstrated that Nar significantly reduced serum TGF-β1 level in Bleomycin-induced lung fiber model, and as a consequence, prevented tumor cell metastasis to lung [22]. Therefore, we suppose that Nar may have a capability to reverse TGF-β1-induced resistance of aspc-1 cells to Gem. Experiments were performed to test the idea. The cells were pre-treated with 5 ng/ml TGF-β1 for 24 h, followed by various concentrations of Gem in the presence or absence of 50 µM Nar for 72 h, the viability was detected by MTT assay. As shown in **Figure 2B**, addition of TGF-β1 increased the resistance of aspc-1 cell to Gem while Nar reversed the TGF-β1-induced resistance. These results suggest that TGF-β1 plays a critical role in the resistance of tumor cells to chemotherapeutical drugs such as Gem.

### Nar suppresses TGF-β1-induced migration and invasion of panc-1 and aspc-1 cells

**Figure 3:**
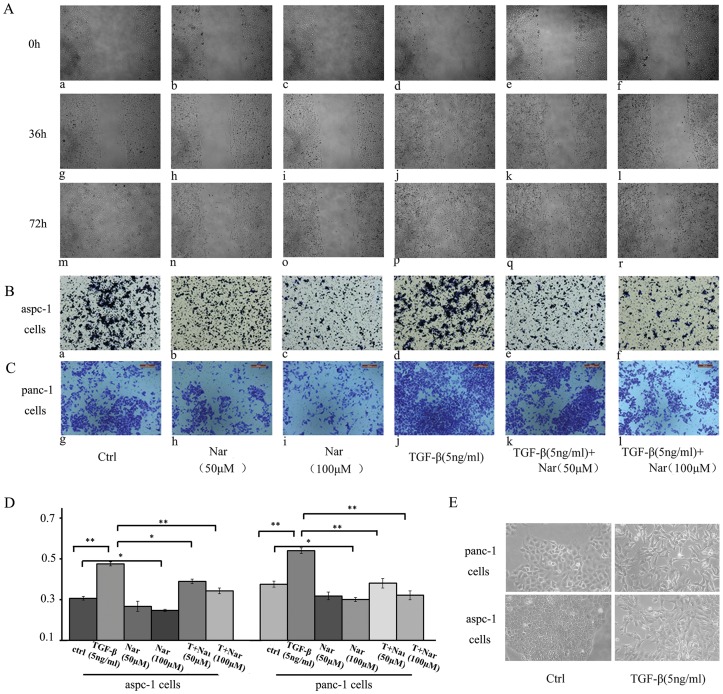
Effect of Nar on migration and invasiveness of aspc-1 or panc-1 cells. (A) Wound healing assay, panc-1 cells grown at 80% confluency. The monolayers were incised with a pipette tip in the central area of the culture. TGF-β1 and Nar was added as indicated times. Photographs were taken using phase-contrast microscopy (magnification at ×100) immediately after the incision and after 36 h and 72 h of treatment. (B and C) Transwell invasion assay (B for aspc-1 cells and C for panc-1cells). In Matrigel-coated cell culture insert, cells were treated as described in Materials and methods. The cells that had invaded to the lower surface of the filter were stained with crystal violet and photographed. (D) Transwell migration assays. In non-Matrigel-coated cell culture insert, to quantify migration, the stained cells were lysed in DMSO and their absorbance was measured. (E) Cell was treated with 5 ng/ml TGF-β1 for 30 h, the phenomenon of EMT was observed by cell morphological changes. These experiments were done triplicate. *p<0.05; **p<0.01; ***p<0.005.

We next determined whether Nar can inhibit TGF-β-induced EMT, which also associates with cellular motility. As expected, morphological changes, including the reduced cell-cell contact and junction, the created fiber and needle types spurious legs, were observed after the cells were treated with TGF-β1 for 30 h (**Figure 3E**), indicating EMT occurrence. In addition, a wound healing assay was performed, the result showed that treatment of panc-1 cells by TGF-β1 for 36 h led to about 80% wound closure (**Figure 3Aj**) compared to the untreated cells (**Figure 3Ag**), indicating that TGF-β1 markedly accelerated EMT process. However, treatment with 50 µM or 100 µM of Nar for 36 h or 72 h significantly inhibited the TGF-β1-induced cell morphological changes and wound closure (**Figure 3Akl *v.s.*** **j**; **Figure 3Aqr *v.s.*** **p**). Moreover, in the absence of TGF-β1, treatment with 50 µM or 100 µM of Nar for 72 h also significantly prevented about 60% wound cells closure (**Figure 3Ano *v.s.*** **m**). Our data demonstrate that endogenous or exogenous TGF-β1 effectively enhances panc-1 cells migration but Nar can reverse this process. Similar results were also observed in aspc-1 cells (data not shown).

Furthermore, we examined the effects of Nar on TGF-β1-induced invasion and migration in aspc-1 and panc-1 cells using transwell assays. The results showed that TGF-β1 treatment markedly enhanced the ability of aspc-1(**Figure 3B** **d *v.s.*** **a**) and panc-1 cells (**Figure 3C** **j *v.s.*** **g**) cross the basement membrane matrix. Panc-1 cells exhibited more aggressive invasive activity than aspc-1 cells responding to TGF-β1 stimulation (**Figure 3C** **j *v.s.*** **Figure 3B** **d**). Interestingly, 50 µM of Nar significantly inhibited the invasive ability of aspc-1(**Figure 3B** **b *v.s.*** **a**) and panc-1 cells (Fig. 3C h. *v.s.* g) in the absence or presence of TGF-β1 (**Figure 3B** **e *v.s.*** **d**; **Figure 3C** **k. *v.s.*** **j**), 100 µM of Nar showed more marked inhibitory effect than 50 µM of Nar in these two cell lines (**Figure 3B** **c *v.s.*** **bf *v.s.*** **e &** **Figure 3C** **i *v.s.*** **h l *v.s*** **k**). In transwell migrate assay, to quantify the migrated cells, we dissolved the cells and then stained with crystal violet in DMSO. Absorbance was measured at 490 nm using a plate reader. Similar results were also obtained (**Figure 3D**). Taken together, we provide strong evidences that Nar can strongly inhibit both basal and exogenous TGF-β1-induced migration and invasion in aspc-1 and panc-1 cells.

### Nar inhibits the process of epithelial to mesenchymal transition (EMT) by reducing expression of mesenchymal markers

**Figure 4:**
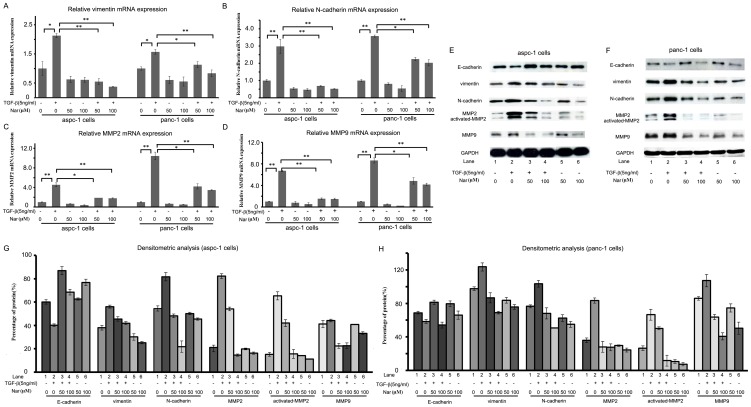
Nar inhibits EMT by suppressing the gene expression of mesenchymal markers. (A–D) Expression of mesenchymal markers of the process of EMT in mRNA level was measured by qRT-PCR. Aspc-1 cells and panc-1 cells were pretreated with 50 µM or 100 µM Nar for 24 h followed by the absence or presence of TGF-β1 for another 24 h (A: vimentin, B: N-cadherin, C: MMP2, D: MMP9). ^#^p<0.05, ^##^p<0.01 *v.s.* Control. ^*^p<0.05, **p<0.01 *v.s.* 5 ng/ml TGF-β1 alone. (E and F). The protein expression of EMT markers was measured by western blot (F for aspc-1 cells and G for panc-1 cells). (G and H) Value of densitometric scan was used to quantify the changes of mesenchymal markers (H for aspc-1 cells, I for panc-1 cells). Data were means ± SD from three independent experiments.

To test whether Nar regulates the production of extracellular matrix and the expression of EMT marker genes in aspc-1 and panc-1 cells in the presence or absence of TGF-β1, real-time RT-PCR analysis was performed. The primers of mesenchymal markers were shown in Table 1. Total RNA was isolated from the cells treated by vehicle or 5 ng/ml TGF-β1 or 5 ng/ml TGF-β1 plus Nar (50 µM, 100 µM). Cells treated with 5 ng/ml TGF-β1 for 24 h led to significant increase in EMT markers mRNAs such as vimentin, N-cadherin, MMP2 and MMP9. Meanwhile, we found that the levels of TGF-β1-induced EMT markers mRNA were significantly reduced in the Nar-treated cells (**Figure 4A, B, C& D**). To evaluate whether the changes of E-cadherin, vimentin, N-cadherin, MMP2 and MMP9 in the gene expression are also associated with their changes in the protein level, western blot was performed after the cells treated by 5 ng/ml TGF-β1 with or without 50 µM Nar (**Figure 4E & F**). The bands were quantified by scanning densitometry using a Bio-Rad model 620Video Densitometer with a 1-D Analyst software package for Macintosh. These data showed that Nar obviously decreased the expressions of vimentin, N-cadherin, MMP2 and MMP9 proteins, and enhanced E-cadherin expression with or without TGF-β1 treatment (**Figure 4G & H**). Additionally, we also detected one band of 68 KDa, an active form MMP2, and one band of 72 KDa, the precursor of MMP2. Our results demonstrated that Nar could inhibit TGF-β1-induced EMT in aspc-1 and panc-1 cells.

**Table 1:** Primers for the present study.

Primers	Sequence (5′ – 3′)		Product size
	Forward primer	Reverse primer	
TβRI	TTACAGCATTGCGGATTA	GATACTTGCGAAGAAAGG	280 bp
TβRII	AGGGAGTGGGTGACATAG	GGAATAAAGAGGACCTGAA	539 bp
Smad2	TCACAGTCATCATGAGCTCAAGG	TGTGACGCATGGAAGGTCTCTC	471 bp
Smad3	GAGTAGAGACGCCAGTTCTACC	CGTTTGGAGAACCTGCGTCCAT	234 bp
Smad7	AATGGCTTTTGCCTCGGACAGC	CACAAAGCTGATCTGCACGGTG	321 bp
Vimentin	GAACGCCAGATGCGTGAAATG	CCAGAGGGAGTGAATCCAGATTA	280 bp
N-cadherin	GAGGAGTCAGTGAAGGAGTCA	GGCAAGTTGATTGGAGGGATG	122 bp
MMP2	ACCCTCAGAGCCACCCCTAA	AGCCAGCAGTGAAAAGCCAG	241 bp
MMP9	TCCCTGGAGACCTGAGAACC	CGGCAAGTCTTCCGAGTAGTT	308 bp
GAPDH	GCAGGGGGGAGCCAAAAGGG	TGCCAGCCCCAGCGTCAAAG	566 bp

### Nar exerts its effect on TGF-β1 signal pathway by regulating Smad3

**Figure 5:**
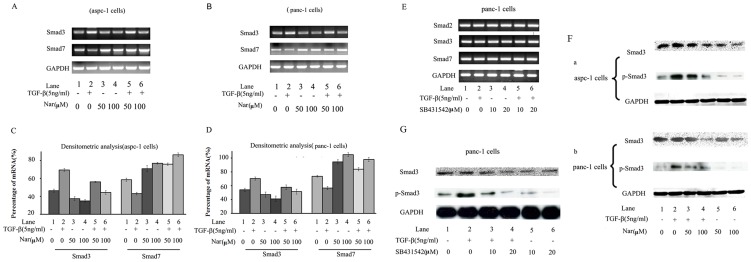
Effects of Nar and SB431542 on the expression of TGF-β/Smads classic pathways related genes. Cells were treated with 50 µM and 100 µM Nar or 10 µM and 20 µM SB431542 for 24 h before with or without 5 ng/ml TGF-β1 addition for 24 h incubation for RT-PCR or 36 h incubation for western blot. (A and B, E) The mRNA level of TβRI, TβRII, Smad2, Smad3 and Smad7 were determined by RT-PCR (A for aspc-1 cells treated by Nar, B for panc-1 cells treated by Nar, E for panc-1 cells treated by SB431542, The result was similar for aspc-1 cells, data not shown). (C and D) Value of densitometric scan was used to quantify the changes of mRNA of Smad3 and Smad7 (C for aspc-1 cells and D for panc-1 cells treated by Nar). (F and G)The level of p-Smad3 was measured by western blot. (F. a for aspc-1 cells and F. b for panc-1 cells treated with Nar, G for panc-1 cells treated with SB431542, The result was similar for aspc-1 cells, data not shown). Data were means ± SD for three individual experiments.

In classic Smad-dependent pathways, TGF-β-induced activation of the receptor complex leads to phosphorylation of Smad2 and Smad3, in cooperation with Smad4 as TGF-β-induced transcription regulators. In contrast, Smad6 and Smad7 inhibit activation of the receptor-regulated Smads [27], [28], [29]. In the present study, we performed RT-PCR to examine the gene expression involved in the regulation of TGF-β signaling. Aspc-1 and panc-1 cells were pretreated with 50 µM or 100 µM Nar for 24 h, followed by treatment with or without TGF-β1 for another 24 h. The mRNA levels of TβRI, TβRII, Smad2, Smad3, Smad4, and Smad7 were monitored by RT-PCR and quantified. The results showed that Nar had no influence on the mRNA levels of TβRI, TβRII and Smad2, even at high concentrations (100 µM) (**Figure S1**). However, treated by TGF-β1, Nar markedly down-regulated the mRNA level of Smad3 (**Figure 5A & C**. **lane 5, 6** ***v.s*** **2**). In the absence of TGF-β1, 50 µM Nar only had a relatively slight influence on Smad3 mRNA and protein level (**Figure 5A & C**. **lane 3, 4** ***v.s*** **1**), but 100 µM Nar obviously decreased Smad3 protein level (**Figure 5F** **ab. lane 6 *v.s*** **1**). These observations indicated that Nar downregulate Smad3 protein level independent of TGF-β1. In the TGF-β signaling pathway, phosphorylation of Smad3 by TβRII is a key step in signal transduction. So we further examined whether Nar can inhibit TGF-β1 induced phosphorylation of Smad3. We found TGF-β1 induced a significant increase in the level of phospho-Smad3 while Nar markedly reduced TGF-β1-induced Smad3 phosphorylation, which effect might be related to the decreased Smad3 protein level induced by Nar. Moreover, this inhibitory effect of Nar was parallel to its dosages used to treat aspc-1 and panc-1 cells (**Figure 5F** **a & b**).

**Figure 6:**
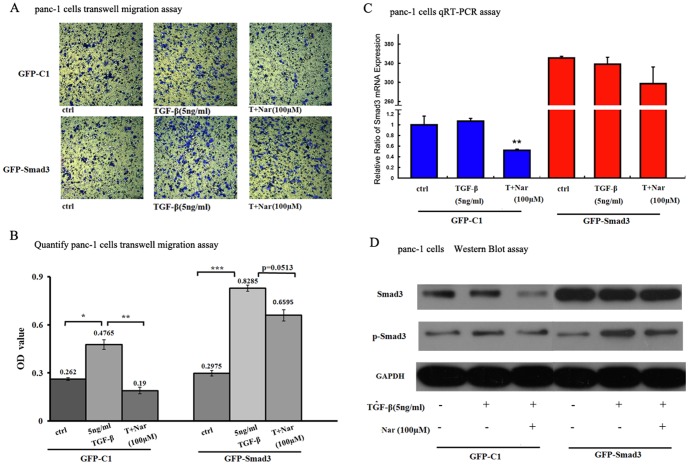
Effects of Smad3 overexpression on the inhibitory effect of Nar on TGF-β1-mediated migration. (A) Transwell migration assay, in non-Matrigel-coated cell culture insert, panc-1 cells were transfected with GFP-Smad3 or GFP-C1 plasmid for 6 h, then washed, changed to complete DMEM with 0.1% DMSO, 100 µM Nar for 24 h, then exposure to 5 ng/ml TGF-β1 or the combination of 5 ng/ml TGF-β1 and 100 µM Nar for next 24 h. As described in Materials and methods. The cells that had invaded to the lower surface of the filter were stained with crystal violet and photographed. (B) Transwell migration assays. In non-Matrigel-coated cell culture insert, to quantify migration, the stained cells were lysed in DMSO and their absorbance was measured. These experiments were done triplicate. *p<0.05; **p<0.01. ***p<0.005. (C) and (D) Panc-1 cells were transfected with GFP-Smad3 or GFP-C1 plasmid for 6 h, then washed, changed to complete medium, 100 µM Nar for 24 h, then exposure to 5 ng/ml TGF-β1 or the combination of 5 ng/ml TGF-β1 and 100 µM Nar for next 24 h for qRT-PCR (C) or western blot (D).

To further demonstrate the role of downregulation of Smad3 on the inhibitory effect of Nar on TGF-β1-mediated phenotype such as migration, we examine whether exogenous overexpression of Smad3 can reverse the inhibitory effect of Nar. We constructed the GFP-Smad3 expression vector using full-length human Smad3 fragment from pCMV5B-Smad3 vector [38] ligated into the GFP-C1 construct (Clonetech). Then we examined the effect of Nar on the migration of panc-1 cells transfected with GFP-Smad3 and GFP-C1 control plasmid. The results showed that both Smad3 mRNA and protein levels were significantly increased in the GFP-Smad3-transfected cells compare to GFP-C1- transfected cells (**Figure 6C & D**), while 100 µM Nar effectively decreased expression of Smad3 both in mRNA and protein levels in the GFP-C1-transfected cells, but failed to significantly reduce the expression of Smad3 in GFP-Smad3-transfected cells in the present of TGF-β1. Importantly, transwell migration assay (**Figure 6** **A & B**) showed that 100 µM Nar obviously inhibited TGF-β1-induced migration in GFP-C1-transfected cells (p<0.01), while exogenous overexpression of Smad3 significantly attenuated the inhibitory effect of Nar on TGF-β1-induced migration. These data indicate that Nar may specially inhibit the endogenous expression of Smad3 and subsequently reduces the phosphorylation of Smad3, thereby, downregulates TGF-β1-induced signal transduction.

On the other hand, SB-431542, a well known specific inhibitor of TβRI, has been shown to inhibit phosphorylation of Smad2/Smad3 [30]. In our study, SB-431542 failed to affect the mRNA expression of Smad3 and Smad7 (**Figure 5E**) but reduced the phosphorylation of Smad3 in panc-1 cells (**Figure 5G**). The similar result was also observed in aspc-1 cells (data not shown). Our data suggest that Nar has a different mechanism from SB-431542 for inhibiting TGF-β-mediated biological effects. Taken together, our results strongly suggest that Nar exerts its effect on TGF-β1 signal pathway mainly through down-regulating Smad3.

## Discussion

EMT promotes tumor cells migration and invasive from the site of origin and diffusion to distant tissues and organs, and also mediates tumor cells developing resistance to chemotherapeutic drugs. This process is triggered by both the autocrine and the paracrine TGF-β signals. Several studies have demonstrated that TGF-β alone or in combination with other growth factors such as EGF plays vital roles in mediating EMT in many malignant tumors including pancreatic cancer [31], [32]. Thus regulating TGF-β signal pathway is effective strategy to control cancer progression.

In the present study, we found that Nar enhanced sensitivity of pancreatic cancer cells to Gem in the presence or absence of TGF-β1. TGF-β1 significantly enhanced the resistance of aspc-1 cells to Gem but Nar completely reversed TGF-β1-induced resistance, suggesting that Nar can block TGF-β1 mediating signal transduction. This finding was also in a good agreement with our previous reports that Nar has abilities to inhibit TGF-β1 secretion and to reduce binding probability of TGF-β1 to its specific receptor TβRII [22], [23]. One of our previous studies demonstrated that Nar enhanced anti-tumor effect of doxorubicin to A549 and MCF-7 caner cells by selectively modulating drug efflux pathways [25]. Other investigators also found that Nar exhibited high antineoplasic activities against the classical MDR subline derived from gastric (EPG85-257), pancreatic (EPP85-181), and colon (HT-29) carcinomas cancer cell lines as well as other derived multidrug-resistant cell lines [33]. These data imply that there may be a link between TGF-β1 signal pathway and multidrug resistance pathways, which to be investigated in future.

In addition, Nar significantly inhibited TGF-β1-induced EMT by decreasing the expression of vimentin, N-cadherin, MMP2 and MMP9 in both mRNA and protein levels. We then examined TGF-β/Smads signal pathway related genes expression in the aspc-1 and panc-1cells, and found that Nar specifically down-regulated Smad3 expression in these two cell lines and exogenous expression of Smad3 could effectively circumvent the inhibitory effect of Nar on TGF-β1-induced EMT phenotype such as migration. Other researchers reported that Smad ubiquitination regulatory factors (Smurfs) induced Smad3 ubiquitination for proteasomal degradation result in disrupting the formation of Smad3 complexes to turn off TGF-β signaling transduction [34]. In our study, the effects of Nar decreasing Smad3 protein level might be independent of TGF-β1, which precise mechanism is still unclear. However, our results clearly indicate that Nar prevents the EMT process of tumor cells by down regulating TGF-β/Smad3 signaling transduction. This finding provides a reasonable explanation why Nar has favorable anti-tumor metastatic efficacy in vivo with slight toxicity to tumor cells in vitro [22], [35]. SB-431542 [36], a small molecular inhibitor of TβIR, had been shown to inhibit TGF-β-induced EMT by regulating the phosphorylation of Smad2/3 in tumor cells, which is consistent with our results. The difference between Nar and SB-431542 in the regulation of TGF-β/Smads signal pathway suggests they might have different biofunctions.

In conclusion, Nar suppresses the migration, invasion and Gem resistance of pancreatic cancer cells by inhibiting TGF-β/Smad3-mediated EMT. These findings reveal that Nar may serve as a new potential agent to prevent tumor progression and metastasis for clinical applications.

## Materials and Methods

### Materials

Naringenin (Nar) was purchased from Shanxi Huike Botanical Development Co. (China). SB-431542 was provided by Sigma-Aldrich Co. (Germany), they were stored as a stock solution in dimethyl sulfoxide (DMSO), which was used after diluting with medium for each assay. The concentration of DMSO did not exceed 0.05% in any assay. Cytokine recombinant human TGF-β1 was purchased from R&D system (Minneapolis, MN, USA) and reconstituted in 10 mM citric acid containing a carrier protein (0.1%BSA), PH 3.0 to a concentration of 2 mg/ml. Gemicitabin (Gem) was provided by Jiangsu Hansoh Pharmaceutical Co.LTD (china). Trizol reagent and SuperScript III Platinum Two-Step qRT-PCR Kit with SYBR Green were obtained from invitrogen™(USA). BCA™ protein Assy Kit was from Thermo scientific (USA). Sepectra™ Multicolor Broad Range protein Ladder and DL1000bp, DL500bp DNA Ladder markers were obtained from TaKaRa Biotechnology Co (Dalian,China).

Protease inhibitor and phosphatase inhibitor cocktail 3 were purchased from Sigma-Aldrich Co.(Germany).Vimentin primary antibody (mouse monoclonal IgG Sc-32322)was provided by Santa Cruz Biotechnology Inc. (Santa Cruz, CA, USA), Cell lysis buffer and Phospho-Smad3(Rabbit mAb IgG Ser423/425), E-cadherin (Rabbit mAb), N-cadherin(Rabbit mAb), GAPDH(Rabbit mAb) primary antibody and second antibodies (Anti-rabbit IgG, HRP-linked Antibody and Anti-mouse IgG, HRP-linked Antibody)was purchased from Cell Signaling Technology Inc. (USA). MMP2 antibody (ab80737, mouse monoclonal IgG), MMP9 antibody (ab76003, Rabbit monoclonal IgG) was purchased from Abcam (Hong Kong). All other reagents were analytically pure.

### Cell culture

Aspc-1 and panc-1 cells were obtained from American Type Culture Collection (Manassas, VA). Cell culture medium and fetal bovine serum were purchased from Invitrogen (Carlsbad, CA). Culture flasks and dishes were from Corning (Corning, NY). Cells were cultured in RPMI 1640 or DMEM medium supplemented with 10% heat-inactivated fetal bovine serum, penicillin (100 U/ml), and streptomycin (100 U/ml), and cultured at 37°C in a humidified atmosphere of 5% CO_2_. Cells were subcultured every 3–4 d. In each assay the drugs were diluted with X-VIVO 15 chemically defined serum-free medium(Lonza, Switzerland)

### Cell Growth Inhibition Assay

Aspc-1and panc-1 cells were plated at a density of 10×10^3^ cells or 3×10^3^ per well in 100 µl RPMI 1640 or DMEM medium in 96-well plates and grown for 24 h, respectively. Cells were exposed to a series of concentrations of Nar for 24 h, 48 h or 72 h, the viability of cells was measured using methylthiazoletetrazolium (MTT) method, as previously described [37]. Briefly, 150 µl MTT solution (0.5 mg/ml in phosphate-buffered saline [PBS]) was added to each well. The plates were incubated for 4 h at 37°C. After incubation, 150 µl of DMSO (Sigma) was added to each well for 10 min at room temperature. Absorbance was measured at 490 nm using a plate reader (Thermo, Erlangen, Germany). The mean percentage of cell survival relative to that of untreated cells was estimated from data of three individual experiments. The concentration of drug at which cell lethaling was 50% was calculated by curve fitting using SPSS software (version 12.0, SPSS, Chicago, IL). For combined with different drugs, after the cells was incubation overnight, the medium was replaced with fresh medium containing (50 µM, 100 µM) Nar alone or combination with TGF-β1 (5 ng/ml) for 24 h and then exposed to Gem for an additional 72 h, The viability of cells was detected by MTT assay.

### Wound healing assay

The scratch was made across the monolayer in aspc-1 cells and panc-1 cells. Cells were cultured on 6-well plates and the confluent cells in culture (80%), cell monolayers were carefully wounded by scratching with a sterile plastic pipette tip. The cells were washed twice with phosphate-buffered saline (PBS), treated with 5 ng/ml TGF-β1 or the combination of 5 ng/ml TGF-β1 and 50 or 100 µM Nar. For each scratch, the Photographs were taken at 0, 36 h, 72 h using an inverted microscope (NIKON ECLIPSE TS100 10×4. Japan) in the same fields. Each experiment was performed at least twice.

### Matrigel invasion and migration assays

Invasion assays were performed using 24-well BD Falcon™ Cell culture insert (8 µm pore size, Becton Dickinson USA). Briefly, cells were seeded into 60 mm disposable cell culture dishes (shanghai sunab Bio-Tech Development Inc. china) and were pre-treated them with 50 or 100 µM Nar for 24 h, then exposure to 5 ng/ml TGF-β1 or the combination of 5 ng/ml TGF-β1 and 50 or 100 µM Nar for next 24 h. The filter was coated with 15 ul of basement membrane matrix (Growth factor reduced, phenol red-free, LDEV-Free, BD Biosciences). The treated cells were trypsinized and resuspended in x-vivo 15 medium and seeded at a density of 3×10^4^ cells or 2×10^4^ per well onto the top chamber. The bottom chamber was filled with 0.6 ml x-vivo15 with 5% FBS contained with samples (control, 5 ng/ml TGF-β1, 50 µM and 100 µM Nar, the combination of 5 ng/ml TGF-β1 and 50 µM or 100 µM Nar) as a chemic attractant. After the assay chambers were incubated for 24 h in a CO_2_ incubator, non-invading cells were carefully removed with a cotton swab. The filter membrane were fixed with cool methanol and stained with crystal violet for 15 min. The cells on the upper surface were gently removed with a cotton swab, and the cells on the lower surface of the filters were shooted under a Leica DM 2500 pathological image and analytical system (Germany) at 50× magnification. All experiments were repeated three times.

Migration assays were performed using 24-well BD Falcon™ Cell culture insert. Briefly, full-length human Smad3 cDNA was amplified from pCMV5B-Smad3 vector [38]. Smad3 fragment was ligated into the GFP-C1 construct (Clonetech) for GFP-C1-Smad3 (GFP-Smad3). Panc-1 cells were cultured in Dulbecco's modified Eagle's medium (DMEM, Gibco) supplemented with 10% fetal bovine serum (Hyclone) at 37°C in a 5% CO_2_. For transfection, Panc-1 cells were incubated with opti-MEM medium (Invitogen) containing GFP-C1 or GFP-Smad3 plasmid for 6 h, then washed, changed to complete DMEM with 0.1% DMSO, 100 µM Nar for 24 h, then exposure to 5 ng/ml TGF-β1 or the combination of 5 ng/ml TGF-β1 and 100 µM Nar for next 24 h, As described in the same conditions as the invasion assays, but non-Matrigel-coated cell culture insert, and the cells which fixed on the lower surface of the filters were dissolved with 150 µL DMSO. Absorbance was measured at 490 nm using a plate reader. The experiments were performed in triplicate and repeated three times.

### Reverse Transcription-Polymerase Chain Reaction (RT-PCR) Assay and Real-time Reverse Transcription-Polymerase Chain Reaction (qRT-PCR)

Total RNAs were isolated from aspc-1 and panc-1 cells using Trizol (Invitrogen, CA, USA). To address the effect of Nar on the mRNA expression of the EMT markers, RNAs were also isolated from aspc-1 and panc-1 cells treated with Nar (50 µM or 100 µM) or the combination of TGF-β1 with Nar respectively. The mRNAs were reversely transcribed to cDNAs by M-MLV reverse transcriptase (Invitrogen, CA, USA). All the primers were purchased from Invitrogen, the sequences of the primer pairs used were listed in **Table 1**. After initial denaturation at 95°C for 5 min, PCR was performed for 30 cycles (30 s at 94°C, 30 s at annealing temperature and 40 s at 72°C) using Taq polymerase (TaKaRa, Japan). Reaction products (20 µl per lane) were electrophoresed in 2% agarose, stained with Goldenview (Biohao Biotechnology Co.Tianjin. china) and photographed. The results were quantitated by scanning densitometry using a Bio-Rad model 620Video Densitometer with a 1-D Analyst software package for Macintosh.

In this study, qRT-PCR was used to detect the expression of smad3 and mesenchymal markers mRNA levels and performed using Platinum® SYBR® Green qPCR SuperMix-UDG (invitrogein) according with the instrument: PCR was initated by a 2 min denaturation at 95°C, followed by 40 cycles of 95°C for 15 seconds, 60°Cfor 30 seconds using an Applied Biosystems 6200HT Sequence Detection System (Foster City, CA, USA). Melting curve analysis refers to instrument documentation. Gene expression was normalized to GAPDH (a housekeeping gene) mRNA expression and presented as fold-change compared to the control experiments for all samples. All assays were performed at least three times and results from one experiment are provided.

### Western Blot

Cells were pre-treated with Nar (50 µM or 100 µM) for 24 h followed by incubation with or without 5 ng/ml TGF-β1 for another 36 h, or panc-1 cells were transfected with GFP-Smad3 and GFP-C1 control plasmid for 6 h, then washed, changed to complete medium, 100 µM Nar for 24 h, then exposure to 5 ng/ml TGF-β1 or the combination of 5 ng/ml TGF-β1 and 100 µM Nar for next 24 h. Cells were harvested, washed, and lysed in ice-cold lysis buffer containing a mixture of protease inhibitors and phosphatase inhibitor, the debris was removed by centrifugation at 13,000 g for 10 min at 4°C. Total protein amount in the extracts was measured using BCA™ protein Assy Kit. Centrifuged lysates from each sample were analyzed by SDS polyacrylamide gel electrophoresis and transferred to a PVDF membrane by semidry transfer (Trans-Blot SD Semi-dry Transfer cell, Bio-Rad, USA). The membranes were blocked for 1 h at room temperature in Tris-buffered saline containing 0.1% Tween 20 (TBST) and 5% non-fat milk. Blots were probed overnight at 4°C with the following primary antibodies overnight (dissolve in TBST containing 5% BSA): anti-Smad3, antiphospho-Smad3, anti-vimentin, anti-N-cadherin, anti-E-cadherin, anti-MMP2, anti-MMP9, and anti-GAPDH antibodies. This was followed by incubation with the appropriate horseradish peroxidase-conjugated secondary antibody at a dilution of 1: 2,000 in TBST containing 5% BSA for 1 h. Detection was achieved by enhanced chemiluminescence (Amersham Pharmacia Biotech) and exposed to film. Filters were quantitated by scanning densitometry using a Bio-Rad model 620Video Densitometer with a 1-D Analyst software package for Macintosh.

### Statistical Analysis

All experiments were carried out at least three times with three independent samples. Statistical analysis was performed using SPSS 12.0 and origin 7.5 program. Data were expressed as the mean ± SD. The differences of the variables between groups were performed with the Student's *t*-test. *P* values less than 0.05 were considered to be statistically significant.

## Supporting Information

Figure S1 Effects of Nar and SB431542 on the expression of TGF-β/Smads classic pathways related genes. Cells were treated with 50 µM and 100 µM Nar or 10 µM and 20 µM SB431542 for 24 h before with or without 5 ng/ml TGF-β1 addition for 24 h incubation for RT-PCR. (A and B, C) The mRNA level of TβRI, TβRII, Smad2, Smad3 and Smad7 were determined by RT-PCR (A for aspc-1 cells treated by Nar, B for panc-1 cells treated by Nar, C for panc-1 cells treated by SB431542).
(TIF)
