# Supplementary figures and images for: Naringenin Decreases Invasiveness and Metastasis by Inhibiting TGF-β-Induced Epithelial to Mesenchymal Transition in Pancreatic Cancer Cells
Source: PLoS One. 2012 Dec 26;7(12):e50956. doi: 10.1371/journal.pone.0050956 (PMC3530567; doi:10.1371/journal.pone.0050956)

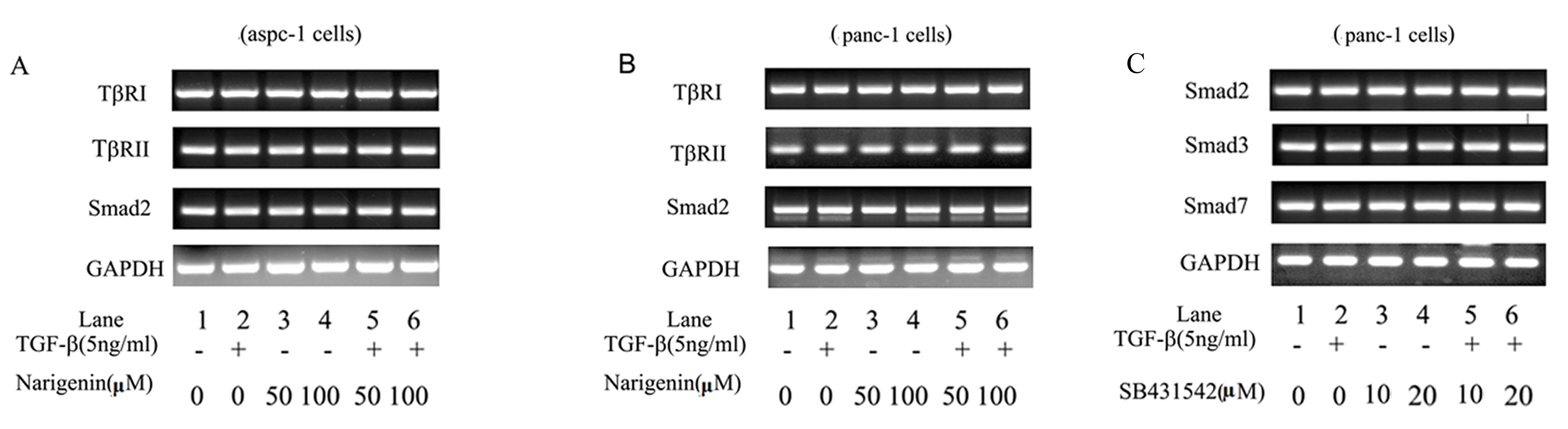

Supplement: Figure S1 — Effects of Nar and SB431542 on the expression of TGF-β/Smads classic pathways related genes. Cells were treated with 50 µM and 100 µM Nar or 10 µM and 20 µM SB431542 for 24 h before with or without 5 ng/ml TGF-β1 addition for 24 h incubation for RT-PCR. (A and B, C) The mRNA level of TβRI, TβRII, Smad2, Smad3 and Smad7 were determined by RT-PCR (A for aspc-1 cells treated by Nar, B for panc-1 cells treated by Nar, C for panc-1 cells treated by SB431542). (TIF) [file pone.0050956.s001.tif]
